# Psychometric evaluation of the PROMIS social function short forms in Chinese patients with breast cancer

**DOI:** 10.1186/s12955-021-01788-8

**Published:** 2021-05-18

**Authors:** Tingting Cai, Qingmei Huang, Fulei Wu, Changrong Yuan

**Affiliations:** School of Nursing, Fudan University, 305 Fenglin Road, Shanghai, 200032 China

**Keywords:** Psychometric analysis, PROMIS, Social function, Breast cancer

## Abstract

**Background:**

The diagnosis of breast cancer and the subsequent treatment undermine patients’ participation in social activities. This study aimed to carry out a cross-cultural adaption and analysis of the construct validity and reliability of the Chinese version of the PROMIS social function short forms in patients with breast cancer.

**Methods:**

This study utilized a cross-sectional research design, and was registered in the Chinese Clinical Trial Registry (ChiCTR2000035439). After a standardized cross-cultural adaption process, a psychometric evaluation was performed of the Chinese version of the PROMIS social function short forms. Using convenience sampling, eligible patients with breast cancer from tertiary hospitals in China were enrolled from January 2019 to July 2020. Participants completed the sociodemographic information questionnaire, the PROMIS social function short forms, the Functional Assessment of Cancer Therapy-Breast, the PROMIS emotional support short form and the PROMIS anxiety short form.

**Results:**

Data were collected from a sample of 633 patients whose mean age was 48.1 years. The measures showed an absence of floor and ceiling effects. Regarding construct validity, the results of confirmatory factor analysis supported the original two-factor structure of the PROMIS social function short forms. In addition, the measures were found to have acceptable known-group validity, measurement invariance, and convergent and discriminate validity. Regarding reliability, the Cronbach’s α was high for all items (> 0.70).

**Conclusion:**

The Chinese version of the PROMIS social function short forms was demonstrated to be a valid and reliable measure for the assessment of social function in Chinese patients with breast cancer. Additional psychometric evaluation is needed to draw firm conclusions.

## Background

Currently, breast cancer is the most common cancer type in women worldwide [1]. Evidence indicates that a higher level of quality of life is related to greater social function [2, 3]. The diagnosis of breast cancer and the subsequent treatment undermine patients’ participation in social activities [4]. In addition, they entail the emergence or worsening of social interaction problems between individuals and their surroundings, which not only affects the rehabilitation process but might also result in treatment interruptions [5]. Furthermore, reduced social participation may be detrimental to patients’ health outcomes [6, 7]. To identify patients with social function issues caused by breast cancer, a measure accounting for the diversity and dynamic nature of social function is required [8].

Social function (or social participation) is conceived as an individual’s involvement in and satisfaction with his or her usual roles in life situations and activities [9]. Social function issues in patients with breast cancer remain an underemphasized area in China, in part because few reliable and valid measures are currently available for incorporation into routine screening in breast cancer care [8]. Routine evaluation of social function that allows the assessment of objective participation performance and subjective satisfaction with participation is needed for patients with breast cancer.

Social function assessment is influenced by several factors, such as cultural factors in the evaluation context. Short and accurate self-report measures are needed to enable the identification of individuals with social function issues and to follow them up. The Patient-Reported Outcomes Measurement Information System (PROMIS) addresses this gap with a series of measures of social health. PROMIS measures contribute to the measurement of a wide range of symptoms, functioning, and well-being outcomes from the patient’s perspective and represent a standardized scoring system with a rigorous and sound methodology covering the physical, mental, and social health domains [10–12]. Within the PROMIS framework, the PROMIS Social Health Workgroup developed measures to evaluate social function by engaging in a series of qualitative and quantitative efforts such as definition formulation, qualitative item reviews, focus groups, cognitive interviews and large-scale testing with a general population sample following standard PROMIS guidelines [9].

The PROMIS social function short forms are a promising alternative to existing instruments for evaluating social function [9]. The measures assess distinct but related aspects of social function and are self-reported measures using only a minimal number of items while maintaining precision [13]. The measures represent great progress in the brief yet accurate assessment of social function for repeated application in situations that require a quick and continuous follow-up assessment and allow for low-burden data capture. The PROMIS social function short forms have been utilized in different cultures and are demonstrated to have adequate psychometric properties in diverse clinical populations [9, 13].

The self-report nature and short valid response method of the PROMIS social function short forms suggest their potential for use as screening tools in the assessment of social function in patients with breast cancer [13]. Considering this, this study aimed to carry out a cross-cultural adaption and analysis of the psychometric properties of the Chinese version of the PROMIS social function short forms in patients with breast cancer.

## Methods

### Design

**Fig. 1:**
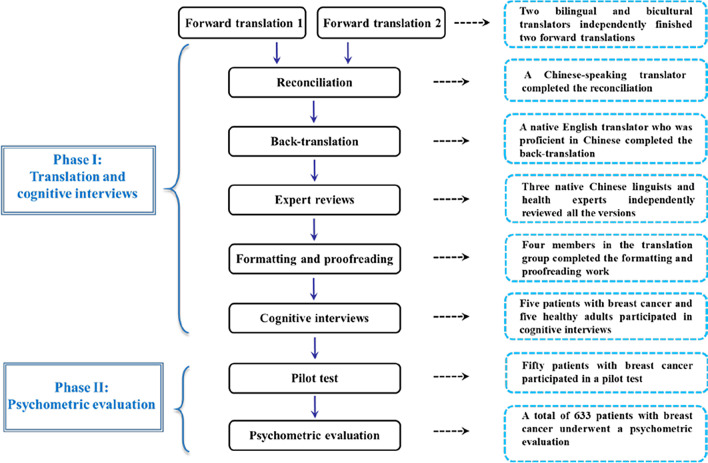
Flow of the multiple standardized phases of the study

This study utilized a cross-sectional research design, and was registered in the Chinese Clinical Trial Registry (ChiCTR2000035439). Two phases were carried out, namely, translation and cognitive interviews, followed by a psychometric evaluation of the Chinese version of the PROMIS social function short forms. Figure 1 presents the flow of the standardized phases of the study.

### Phase I: translation and cognitive interviews

The PROMIS Health Organization authorized our research group to translate the original English version of the PROMIS social function short forms into simplified Chinese. A standardized transcultural procedure was carried out by strictly following the Functional Assessment of Chronic Illness Therapy translation method (FACITtrans), which is an international guideline for translation procedures recommended by the PROMIS Statistical Center [14]. The translation involved multiple forward translations, reconciliation, back-translation, independent expert reviews, formatting, and proofreading following recommended guidelines. All the translators have a master's degree or above; one is a linguistics expert and two are Chinese-Canadian researchers who are native English speakers and have a good command of Chinese. First, two bilingual and bicultural translators from China and Canada independently completed two forward-translated versions of the measures into simplified Chinese. Both of these translators had a background in linguistics. Following this, a Chinese-speaking translator completed a reconciliation of the two versions. Back-translation was then performed by a native English translator who was proficient in Chinese. Subsequently, three native Chinese linguists and health experts were recruited to independently review all the versions to select the most appropriate one or to provide alternative translations for an optimal version. Finally, the translation project manager of our research group completed the formatting and proofreading work with the help of a language coordinator and two proofreaders to combine all previous versions and made necessary item modifications.

Therefore, the PROMIS social function short forms was determined by consensus. The measures were then validated in Chinese-speaking adults to compare the item equivalence between the Chinese version and the original English version. Participants were asked to report whether there were incomprehensible items and then provide a more appropriate expression. Five patients with breast cancer and five healthy adults were recruited for cognitive interviews on the items and the response options. The measures were further modified based on participants’ feedback to make them more comprehensible and acceptable and were reviewed by the PROMIS Health Organization. The prefinal version was pilot-tested in a sample of 50 Chinese patients with breast cancer and subsequently revised.

### Phase II: psychometric evaluation

Before widely applying the PROMIS social function short forms in clinical settings, a multicenter cross-sectional study was conducted to assess the psychometric properties in terms of construct validity and reliability following the practices recommended by Consensus-based standards for the selection of health measurement instruments (COSMIN).

#### Setting and sample

Using a convenience sampling method, participants from the breast care wards of tertiary hospitals in Shanghai Province were recruited from January 2019 to July 2020. The inclusion criteria for patients were as follows: (a) aged 18 or older, (b) had a diagnosis of stage I to IV breast cancer, (c) received medical treatment for breast cancer, (d) had an adequate understanding of Mandarin, and (e) signed an informed consent form. Patients with psychiatric illness, cognitive impairment or diagnosis of another cancer type were excluded. According to the recommendation of the PROMIS National Center, three to ten respondents should participate in cognitive interviews for each item [14]. Because there are eight items in the PROMIS social function short forms, we chose five patients with breast cancer and five healthy adults for cognitive interviews. Regarding the psychometric evaluation, the ratio of cases to variables was > 20:1 (633:8) in this study, which is over the recommended rule of thumb value (5–10:1) [15]. The sample size was sufficient to perform stable and precise model estimation by confirmatory factor analysis (CFA) [15].

### Measures

#### Sociodemographic information questionnaire

A sociodemographic information questionnaire was developed to collect sociodemographic and clinical data regarding age, marital status, childbearing history, religion, educational background, menstrual status, living style, employment status, monthly family income, health insurance, and medical treatment. Sociodemographic data were self-reported by the patients, while clinical data were obtained from their medical records by trained nurse researchers.

#### PROMIS social function short forms

The PROMIS social function short forms comprise two subscales, namely, the PROMIS Ability to Participate in Social Roles and Activities short form and the PROMIS Satisfaction with Social Roles and Activities short form, of varying lengths (4-, 6-, and 8-item short forms); they assess limitations and satisfaction with social function, respectively [16]. The PROMIS social function short forms in all three lengths have been reported to be highly reliable across mild to severe levels of clinical severity and sensitive to differences in self-reported social function [17]. Therefore, we selected the shortest version of the measures (4-item short forms) to reduce the burden on the respondents. The total raw score ranges from 8 to 40, with high scores representing a high level of social participation or satisfaction with social participation. Subsequently, the scores were normalized according to a mean standardized T-score metric, with a mean of 50 and a standard deviation of 10 representing the average level of the general US population [18, 19].

#### Functional assessment of cancer therapy-breast

The Chinese version of the Functional Assessment of Cancer Therapy-Breast (FACT-B) is a 36-item multidimensional scale specifically designed to assess quality of life in the past 7 days in patients with breast cancer [20]. The FACT-B was used in the present study as an indirect measurement to explore the convergent validity of the PROMIS social function short forms. The scale comprises five subscales: physical, social/family, emotional, functional well-being, and the breast cancer-specific subscale [21]. Each item is evaluated on a 5-point Likert-type scale from 0 (“not at all”) to 4 (“very much”). The overall score is the summary score of the subscales; the total score ranges from 0 to 144, and higher scores indicate better quality of life [22, 23]. The Chinese version of the FACT-B has acceptable psychometric properties in Chinese patients with breast cancer [24, 25]. The Cronbach’s α of the scale was 0.93 in this study.

#### PROMIS emotional support short form

The Chinese version of the 4-item PROMIS emotional support short form was utilized to explore the convergent validity of the PROMIS-social function short forms. The responses are scored within a seven-day recall period with a five-point Likert scale (1 = never, 2 = rarely, 3 = sometimes, 4 = often, 5 = always) [26]. The total raw score ranges from 8 to 40, with higher scores indicating better emotional support [27]. The scores are reported as T scores (with a mean of 50 and a standard deviation of 10) [9]. The scale has been validated in Chinese patients with breast cancer [28]. The Cronbach’s α of the scale was 0.92 in this study.

#### PROMIS anxiety short form

The Chinese version of the 8-item PROMIS anxiety short form was utilized to examine the discriminant validity in this study. The items use a 7-day time frame and a 5-point rating scale (1 = never, 2 = rarely, 3 = sometimes, 4 = often, and 5 = always) [28]. The total score of the scale ranges from 8 to 40, with higher scores indicating greater anxiety [29]. Raw scores are transformed on a T metric (mean = 50, SD = 10). The Cronbach’s α of the scale was 0.93 in the current study.

### Procedures

The ethics committee of the Institutional Review Boards of Fudan University Cancer Hospital (no 1810192-22) and Fudan University Zhongshan Hospital (no 2020-076R) reviewed and approved this study. Eligible patients who met the inclusion criteria were invited to participate after a review of the medical records. The participants gave their voluntary consent to be involved with the help of their nurses during hospitalization. Data were collected by trained nurse researchers at each study site. All the participants were informed about the purpose and procedures of the study. In addition, participants were informed of the voluntary nature of participation, participants’ rights, and the confidentiality of the data. All participants gave formal written consent to participate. Participants could choose to complete the survey either on paper or using web-based questionnaires based on their preferences. The participants were required to return the questionnaire immediately after completion. Consenting participants completed the sociodemographic information questionnaire, the PROMIS social function short forms, the Functional Assessment of Cancer Therapy-Breast, the PROMIS emotional support short form and the PROMIS anxiety short form. Data were checked for random responding.

### Statistical analysis

The statistical analyses were performed with IBM SPSS version 21.0 and AMOS version 23.0. Descriptive statistics were calculated for the sociodemographic data and to determine the distribution of items. A floor effect refers to the proportion of patients with the lowest raw scores, whereas a ceiling effect refers to the proportion of patients with the highest raw scores, with a proportion greater than 15% being considered indicative of a floor or ceiling effect [30].

The construct validity of the measures was examined. Two constructs, limitations and satisfaction with social function, were supposed to be found according to the conceptual framework developed by the authors of the PROMIS social function short forms [16]. CFA was performed to identify the underlying factor structure of the Chinese version of the PROMIS social function short forms, and a CFA model with a two-factor structure was expected to be supported. The measures were treated as ordered categorical variables in the CFA analysis. To examine the goodness of model fit, indices including the χ^2^/degree of freedom (χ^2^/df), goodness-of-fit index (GFI), comparative fit index (CFI), Tucker–Lewis index (TLI), incremental fit index (IFI), and root mean square error of approximation (RMSEA) were included. An acceptable CFA model should have a χ^2^/df < 3; a GFI, CFI, TLI and IFI > 0.9; and a RMSEA < 0.08 [31, 32]. Items with a factor loading equal to or higher than the criterion of 0.4 were retained [33].

Considering the results of previous studies, the social function of patients with breast cancer was expected to differ significantly by employment status [34, 35]. Therefore, known-group validity was evaluated by comparing T-scores between patients reporting different employment statuses using the analysis of variance.

To test whether the measures provided biased results across different populations, differential item functioning (DIF) was examined for each item in the PROMIS social function short forms. DIF analyses were performed to test the measurement invariance and identify whether patients with the same trait from different groups have different probabilities of giving certain response to items [36]. Items with significant DIF indicate measurement bias [36]. Therefore, measurement invariance was evaluated by considering DIF of the PROMIS social function short forms due to age and education.

Convergent and discriminant validity were examined by testing the correlations between similar and dissimilar traits. Pearson correlation coefficients were utilized in this study, with values of 0–0.30 representing negligible correlation; 0.30–0.50 indicating weak correlation; 0.50–0.70 indicating moderately strong correlation and above 0.70 indicating strong correlation [37]. Convergent validity is supported when the scores of measures in a similar domain are correlated, but not so strongly as to be redundant (r between 0.40 and 0.80) [38]. Discriminant validity is established when correlations between scores of different traits are low (r < 0.30) [38]. We hypothesized that the social function level of patients with breast cancer would be positively correlated with their degree of quality of life and emotional support. Therefore, it was hypothesized that the correlations between the scores of the PROMIS social function short forms and those of the Functional Assessment of Cancer Therapy-Breast and the PROMIS emotional support short form would be significant since all these measures focus on social health. Conversely, the correlations between the PROMIS social function short forms score and the PROMIS anxiety short form score should be negligible since the measures are of dissimilar constructs.

The reliability of the measures was evaluated by Cronbach's α coefficient, split-half reliability, and item-to-total correlations. Minimally acceptable reliability was specified as greater than 0.70 [39, 40]. For all statistical analyses, a probability of 0.05 was used to indicate statistical significance.

## Results

### Descriptive statistics

A total of 750 questionnaires were distributed. Ninety-nine eligible patients did not consent to participate, while 651 agreed to be involved. The major reason for refusal was being overwhelmed with their cancer treatment or their family members not agreeing with their participation. In addition, 18 questionnaires were excluded for being incomplete. The final data were obtained from 582 paper questionnaires and 51 web-based questionnaires. Therefore, psychometric analysis of the PROMIS social function short forms was performed with a sample of 633 participants. The average age of the respondents was 48.1 years, with a range of 23–76 years (SD = 9.97). Most respondents reported that they were married (94.16%), had a childbearing history (98.39%), were premenopausal (52.76%), had no religion (92.10%), finished secondary school (31.75%), lived with family (94.15%), were unemployed (45.82%), had a monthly family income of less than ¥3000/$450 (52.61%), had employee health insurance (53.87%), and underwent chemotherapy (92.58%) (Table 1).

**Table 1:** Demographic and clinical characteristics of the study sample (N = 633)

Variables	Frequency (Percent)
Age (Mean ± SD)	48.07 ± 9.97
Marital status	
Single	13 (2.05)
Married	596 (94.16)
Divorced	14 (2.21)
Widowed	10 (1.58)
Childbearing history	
Yes	615 (98.39)
No	18 (1.61)
Menstrual status	
Premenopausal	334 (52.76)
Postmenopausal	299 (47.24)
Religion	
Yes	50 (7.90)
No	583 (92.10)
Education background	
Primary school or below	157 (24.80)
Secondary school	201 (31.75)
High school	129 (20.38)
University or above	146 (23.07)
Lifestyle	
Living alone	18 (2.84)
Living with family	596 (94.15)
Living with others	19 (3.01)
Current employment	
Employed	92 (14.53)
Medical leave	132 (20.85)
Unemployed	290 (45.82)
Retired	119 (18.80)
Monthly family income	
≤ ¥3000 ($450)	333 (52.61)
¥3000-¥9000 ($450-$900)	274 (43.29)
> ¥9000 ($900)	26 (4.10)
Medical insurance	
Free medical insurance	4 (0.63)
Employee health insurance	341 (53.87)
Rural health insurance	257 (40.60)
Without health insurance	31 (4.90)
Medical treatment	
Postoperative stage	6 (0.95)
Chemotherapy	586 (92.58)
Radiotherapy	17 (2.69)
Targeted therapy	8 (1.26)
Endocrine therapy	2 (0.32)
Combination of therapies	14 (2.20)

Table 2 provides an overview of the proportion of respondents who achieved the lowest or highest raw scores for each item. No floor or ceiling effects were found for the PROMIS social function short forms (Table 3). We transformed theta scores into T scores. The average T scores for the PROMIS Ability to Participate in Social Roles and Activities short form and the PROMIS Satisfaction with Social Roles and Activities short form were 51.49 ± 9.86 and 50.36 ± 9.92, respectively; both scores are average.

**Table 2:** Item-level descriptive analysis

Item	Mean	SD	Response of “1”		Response of “5”		Skewness	Kurtosis
			n	%	n	%		
Ability to participate in social roles and activities 01	2.95	0.90	41	6.48	35	5.53	0.01	0.48
Ability to participate in social roles and activities 02	2.89	0.93	43	6.79	36	5.69	0.14	0.19
Ability to participate in social roles and activities 03	2.94	0.92	41	6.48	35	5.53	0.04	0.20
Ability to participate in social roles and activities 04	2.95	0.90	41	6.48	30	4.74	− 0.44	0.37
PROMIS satisfaction with social roles and activities 01	3.15	1.05	35	5.53	63	10.00	− 0.06	− 0.45
PROMIS satisfaction with social roles and activities 02	3.19	1.01	26	4.11	62	9.80	− 0.04	− 0.49
PROMIS satisfaction with social roles and activities 03	3.21	1.03	34	5.37	63	10.00	− 0.18	− 0.44
PROMIS satisfaction with social roles and activities 04	3.18	1.03	31	4.90	65	10.27	− 0.13	0.47

**Table 3:** Floor and ceiling effects of the PROMIS social function short forms

Short form	Floor, N (%)	Ceiling, N (%)
PROMIS ability to participate in social roles and activities short form (raw sum score)	26 (4.11)	23 (3.63)
PROMIS satisfaction with social roles and activities short form (raw sum score)	18 (2.84)	46 (7.27)

### Construct validity

**Fig. 2:**
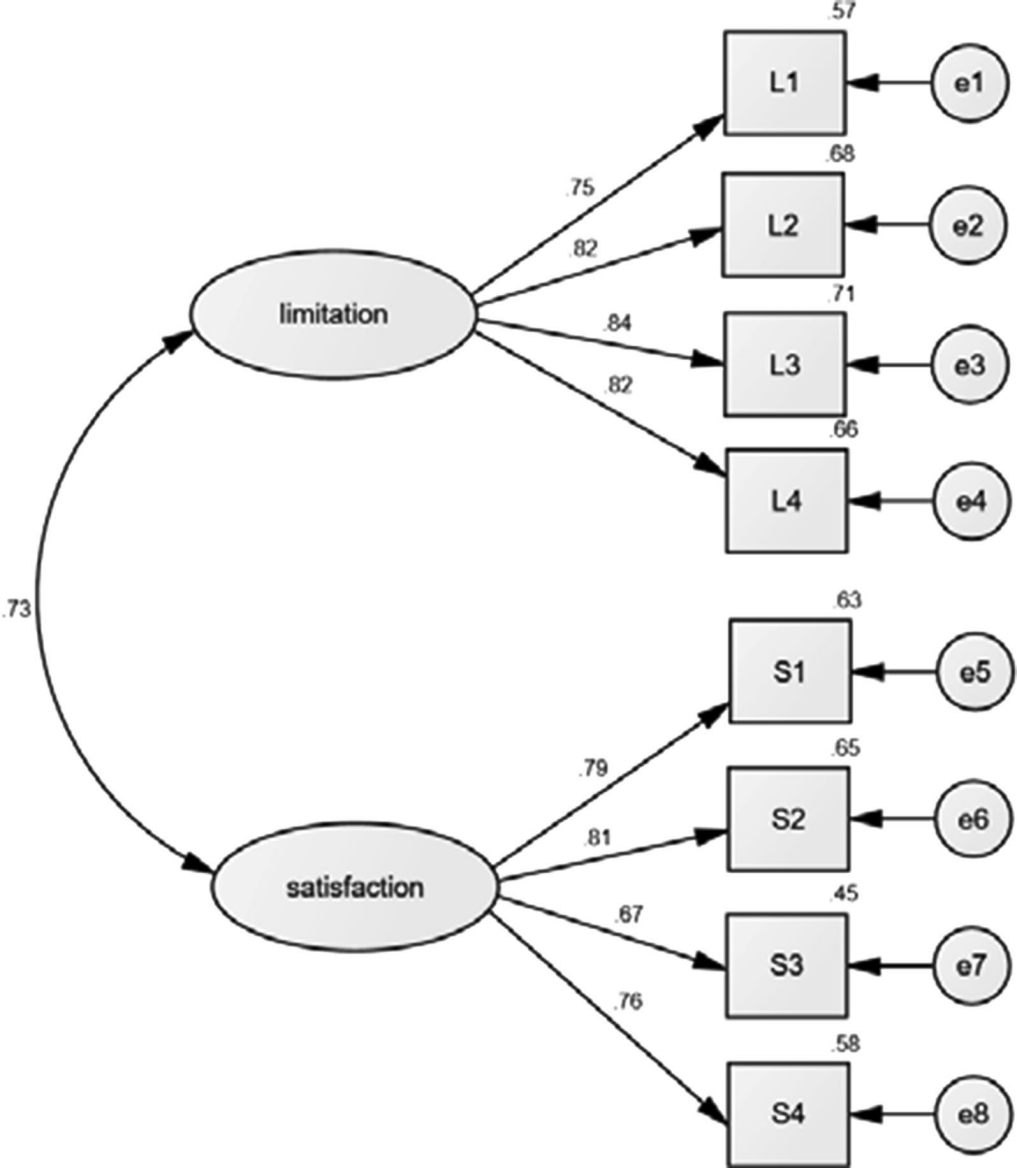
Confirmatory factor analysis model of the PROMIS social function short forms

We examined the two-factor solution of the CFA. All factor loadings in the two-factor CFA model of the 8 items were above the standard of 0.4 (Fig. 2). The goodness of fit of the two-factor model was acceptable: χ^2^/df = 2.133, *P* < 0.001, RMSEA = 0.052, GFI = 0.931, CFI = 0.939, TLI = 0.910, and IFI = 0.923. In addition, there were positive correlations between the two constructs of social function (*P* < 0.05). The variable loadings on limitation of social function ranged from 0.75 to 0.84; the loadings on satisfaction with social function ranged from 0.67 to 0.81. The results supported a two-factor structure of the Chinese version of the PROMIS social function short forms in patients with breast cancer.

To examine the known-group validity of the PROMIS social function short forms, the scores were compared across patients with different employment statuses. The results provided evidence that social function scores were significantly different in employed and unemployed patients with breast cancer, with employed individuals reporting higher scores than those who were unemployed, indicating acceptable known-group validity (Table 4).

**Table 4:** Known-group validity of the PROMIS social function short forms

Known groups	PROMIS ability to participate in social roles and activities		PROMIS satisfaction with social roles and activities	
	Mean (SD)	*P*	Mean (SD)	*P*
Employed	49.67 (12.20)		51.91 (12.14)	
Unemployed	50.35 (8.14)	< 0.001	48.99 (9.18)	< 0.001

Measurement invariance was evaluated by examining DIF. Specifically, patients of different ages (18–39 years, 40–59 years, and ≥ 60 years) and educational backgrounds (primary school or below, secondary school, high school, and university or above) were compared to identify whether there was DIF in the items. No significant DIF was found for any item, which showed that the measures were invariant across patients with different sociodemographic characteristics (Table 5).

**Table 5:** Differential item functioning parameter estimates for the PROMIS social function short forms

Items	DIF test *P* value	
	Age group	Education group
Ability to participate in social roles and activities 01	0.466	0.796
Ability to participate in social roles and activities 02	0.941	0.851
Ability to participate in social roles and activities 03	0.703	0.813
Ability to participate in social roles and activities 04	0.732	0.952
Satisfaction with social roles and activities 01	0.223	0.125
Satisfaction with social roles and activities 02	0.075	0.099
Satisfaction with social roles and activities 03	0.122	0.289
Satisfaction with social roles and activities 04	0.088	0.070

Regarding convergent validity and discriminant validity, in accordance with our hypotheses, the PROMIS social function short forms presented a significant correlation with the PROMIS emotional support short form and the Functional Assessment of Cancer Therapy-Breast. On the other hand, negligible correlations were found between the scores of the PROMIS social function short forms and the PROMIS anxiety short form. Therefore, the results indicated that higher scores on the PROMIS social function short forms were related to higher emotional support and quality of life scores but not significantly correlated with anxiety scores. The largest correlation was between the scores of the PROMIS Ability to Participate in Social Roles and Activities and PROMIS emotional support short form (r = 0.54, *P* < 0.05), which was a moderately strong correlation. The weakest correlation emerged between the scores of the PROMIS Ability to Participate in Social Roles and Activities and the PROMIS anxiety short form (r = 0.08, *P* < 0.05), suggesting a negligible correlation (Table 6).

**Table 6:** Convergent validity and discriminant validity of the PROMIS social function short forms

Short form	PROMIS emotional support short form		Functional assessment of cancer therapy-breast			PROMIS anxiety short form	
	*r*	*P*	*r*	*P*		*r*	*P*
PROMIS ability to participate in social roles and activities	0.54	< 0.001	0.359	< 0.001	0.08		0.045
PROMIS satisfaction with social roles and activities	0.48	< 0.001	0.317	< 0.001	− 0.19		< 0.001

### Reliability analysis

Regarding the reliability analysis, the internal consistency coefficients, Guttman split-half coefficient, and item-to-total correlations were calculated. The Cronbach’s α values and split-half coefficients of the PROMIS Ability to Participate in Social Roles and Activities short form and the PROMIS Satisfaction with Social Roles and Activities short form were above the standard of 0.70. In addition, the item-total correlations also indicated the acceptable reliability of the measures (Table 7).

**Table 7:** Reliability of the PROMIS social function short forms

Short form	Cronbach’s α	Split-half coefficient	Item-total correlations
PROMIS ability to participate in social roles and activities short form	0.88	0.85	0.68–0.75
PROMIS satisfaction with social roles and activities short form	0.84	0.78	0.69–0.77

## Discussion

To the best of our knowledge, this study represents the first application of the PROMIS social function short forms in the Chinese cancer context. Following the PROMIS guidelines, a rigorous approach was used to translate the original English version of the PROMIS social function short forms into a validated and culturally sensitive simplified Chinese version. Subsequently, we performed a cross-cultural adaption and psychometric testing of the PROMIS social function short forms in Chinese patients with breast cancer. The findings demonstrated the potential of the measures to reduce patient burden in addition to providing adequate reliability and construct validity in patients with breast cancer.

Regarding reliability, all items were above the minimal acceptable criterion of 0.70, comparable with the original English version and suggesting acceptable reliability of the measures [19, 41]. Although no floor or ceiling effects were found, the floor effects in this study (4.11%, 2.84%) were higher than those reported by Carlozzi et al. (1.30%, 1.30%) [41]. Additionally, the ceiling effects (13.50%, 12.70%) were higher than those found by Carlozzi et al. (3.63%, 7.27%). The average T scores for the PROMIS Ability to Participate in Social Roles and Activities short form and the PROMIS Satisfaction with Social Roles and Activities short form were 51.49 ± 9.86 and 50.36 ± 9.92, respectively, higher than the scores reported by Carlozzi et al. (49.80 ± 8.60 and 47.80 ± 8.30) [41]. Therefore, compared to the patients with traumatic brain injury investigated by Carlozzi et al. [41], patients with breast cancer might have better social function.

The results of CFA supported the original two-factor structure of the PROMIS social function short forms, consistent with the theoretically expected domains of limitation of and satisfaction with social function [16, 18]. The positive effect of employment status for patients with breast cancer has been proven in recent studies, in which work adjustments were a protective factor for occupational rehabilitation after the cancer diagnosis [42–44]. Consistent with our hypotheses, the PROMIS social functions short forms performed well in differentiating patients with different employment statuses, since employed patients reported better social function than unemployed individuals. The PROMIS measures are expected to use items without measurement bias across individuals who differ in terms of gender, age, and education [18]. Male breast cancer is a rare disease with an incidence of approximately 1% in China [45]. We failed to recruit male patients with breast cancer in this study. Since all participants in this study were women, we explored DIF only for the items regarding education and age. In accordance with the Dutch version of the PROMIS social functions short forms, patients with different ages and education levels interpreted the meaning of the items in a similar way, and the score differences were not due to group differences but to actual differences in social function, supporting the use of the measures in Chinese patients with breast cancer.

The convergent validity was supported by the strong correlations between the PROMIS social function short forms and measures of similar constructs. On the other hand, discriminant validity was confirmed by negligible correlations between the social function measures and measures of dissimilar constructs. The Functional Assessment of Cancer Therapy-Breast is a quality-of-life measure rather than a social health measure. Therefore, we hypothesized that the PROMIS Social functions short forms scores would correlate strongly (r > 0.50) with the PROMIS emotional support short form than the Functional Assessment of Cancer Therapy-Breast. The measures were expected to be negligibly correlated with the PROMIS anxiety short form. The correlation results indicated that the greater the social function was, the greater the level of emotional support and quality of life, suggesting that the PROMIS social function short forms were compatible with social health-related measures but had low correlations with dissimilar measures. The findings were in line with a previous study showing the strong correlations of both the English and Spanish versions of the PROMIS social function short forms with social health-related measures such as the Functional Assessment of Cancer Therapy-General and the MOS 36-item Short Form Health Survey [27].

Given the results, the PROMIS social function short forms were acceptable for the clinical assessment of social function in patients with breast cancer. Having social function measures with sufficient psychometric properties is an important step for healthcare professionals to identify patients’ social function issues and implement targeted interventions. Future studies are advisable to evaluate how the measures work in Chinese patients with other cancer types.

### Limitations

This study has several limitations. First, our study is limited in its generalizability due to the convenience sampling of patients with breast cancer from tertiary hospitals. Only female patients were enrolled due to the very low incidence of male patients with breast cancer in China. Failure to recruit male patients is one of the limitations of this study. The sample may therefore not be representative of the total population of Chinese patients with breast cancer. In addition, data on test–retest reliability, responsiveness evaluations and validity of reference assessments for hypothesis testing are warranted to further examine the psychometric properties of the measures.

## Conclusion

This study indicates that the Chinese version of the PROMIS social function short forms has acceptable reliability and construct validity in patients with breast cancer. Further psychometric evaluation of some domains is warranted to draw firm conclusions.

## Data Availability

All data presented in this paper are available from the corresponding authors on reasonable request.
